# Pre-existing osteoporosis and serum vitamin D levels in patients with distal radius fractures: are we missing something?

**DOI:** 10.1007/s00402-024-05199-4

**Published:** 2024-02-02

**Authors:** Steffi S. I. Falk, Meike Richter, Josephine Schröder, Sina Böhme, Thomas Mittlmeier

**Affiliations:** Clinic of Trauma, Hand and Reconstructive Surgery, University of Rostock, Schillingallee 35, 18055 Rostock, Germany

**Keywords:** Treatment gap, Distal radius, Vitamin D, Osteoporosis, Diagnosis gap, Elderly

## Abstract

**Introduction:**

Given the significant therapeutic gap for osteoporosis, this study aims to investigate the most common osteoporosis-related fracture. The analysis will also consider patients’ serum vitamin D levels and the indications for basic osteoporosis diagnostic tests and osteoporosis therapy prior to fracture.

**Materials and methods:**

This prospective clinical trial included patients with distal radius fractures who underwent surgery at our hospital between 1 April 2021 and 7 April 2022. Blood samples were taken from all participants and existing risk factors for osteoporosis were recorded. In addition, the indication for a guideline-based osteoporosis diagnosis was assessed and the risk of another future fracture with FRAX^®^ was calculated. This information was used to decide whether there was an indication for specific osteoporosis therapy.

**Results:**

A diagnosis gap of 53% and a treatment gap of 84% were identified among the 102 patients investigated. The patients’ ages ranged from 46 to 91 years, with an average vitamin D level of 57 nmol/l, which was below the recommended level of 75 nmol/l. It was noted on a monthly basis that the vitamin D level (without substitution) never exceeded the recommended value of 75 nmol/l in any month. Three-quarters of patients had indications for a baseline osteoporosis diagnosis, yet less than 50% received one. According to FRAX^®^ data, 57% of patients had indications for specific osteoporosis treatment before experiencing the fracture.

**Conclusion:**

Even without a previous distal radius fracture, many patients are in need of osteoporosis diagnosis or treatment. Our research suggests that patients with distal radius fractures should have their vitamin D levels checked via a blood test and be evaluated for osteoporosis. As endogenous vitamin D levels are often inadequate, year-round vitamin D supplementation should be considered for the prevention of osteomalacia and as a basis for the treatment of osteoporosis.

**German clinical trial register ID:**

DRKS00028085.

## Introduction

The distal radius fracture is the most common fracture in humans and its incidence is increasing [1, 2]. This fracture reflects early changes in bone and muscle fragility and is the most common major osteoporotic fracture. It is thus recognized as the index fracture of osteoporosis with an increased risk of subsequent fractures, notably hip fractures [3, 4].

Current data show a large treatment gap for osteoporosis in Germany and Europe. According to the latest data from the SCOPE study [5], this gap has increased from an average of 54% in 2010 to 74% today. This is associated not only with rising healthcare costs, but also with an increase in the incidence of osteoporosis-related fractures. Osteoporosis-related fractures, in particular, represent a significant risk of need for care and lead to a decline in the quality of life of our patients.

The question is whether, and if so to what extent, there is a treatment gap prior to the most common osteoporosis-related fracture, the fracture of the distal radius. A treatment gap would provide a starting point for prevention to get closer to the goal of starting to treat osteoporosis before the first fracture occurs. Knowing that a fracture of the distal radius is often the first clinical fracture to occur in osteoporosis, the diagnosis recommended in the guideline for existing risk factors, such as age or previous diseases, also comes into focus. Without such a diagnosis, it is unlikely that osteoporosis will be diagnosed, and it is highly unlikely that any treatment will be given. This is why we also need to know whether there is the same gap in the diagnosis of osteoporosis as there is in treatment, and what the status of bone metabolism is in these patients.

Vitamin D is one of the most important key elements in bone metabolism. It regulates calcium and phosphate metabolism, thereby promoting bone hardening. A vitamin D deficiency leads to a disturbance in bone metabolism. As a result, bone becomes demineralized and can contribute to the development of osteoporosis.

In the literature, the requirement for older adults is 800–1000 IU vitamin D per day [6, 7]. For sufficient endogenous production of vitamin D in northern Germany for a person of 70 years, outside the winter months, an average of 40 min of face and arm exposure to the midday sun is required in March, down to 24 min in September and 12 min in June and July [8, 9]. The times are calculated for exposure to the sun on bare skin without any sunscreen.

With the advent of skin cancer prevention, the question now arises as to whether sufficient endogenous synthesis is achieved in countries such as Germany. The study presented here focuses on patients with distal radius fractures—a particularly vulnerable group with regard to disorders of bone metabolism.

The hypotheses investigated here are (i) even before a fracture of the distal radius, there is a huge gap in the treatment of osteoporosis as well as in its diagnosis, and (ii) sufficient vitamin D synthesis only occurs during the summer months and only in young adult patients. In older patients, regardless of gender, endogenous synthesis may be inadequate and substitution may be required.

## Materials and methods

The data presented here originate from a prospective clinical study of patients who underwent surgical treatment of distal radius fractures at our hospital corresponding to a level-one trauma center. Patients aged 45 years or older with unstable distal radius fractures were prospectively included in this analysis from April 2021 for a period of one year; patients with pathological fractures or renal insufficiency were excluded. In addition, the injury had to be the result of a low-energy trauma, e.g., via tripping, slipping or falling from a standing position or low level.

Peripheral venous blood samples were collected from all participants and analyzed for parameters with an influence on vitamin D or bone metabolism (see Table 1). The calcium was then corrected for the albumin level according to the formula of Payne et al. [10]. To avoid bias due to vitamin D intake, patients were interviewed and then divided into 3 groups (with self-prescribed, without and with physician-prescribed vitamin D supplementation). 25-hydroxyvitamin D was measured by chemiluminescence microparticle immunoassay (CMIA) in the hospital laboratory.

**Table 1:** Characteristics and determinants of vitamin D or bone metabolism in blood serum with reference to the standard ranges valid in the laboratory at our hospital

Characteristic	Abbreviation	Normal range
25-hydroxyvitamin D	Vit. D	75–100 nmol/l
Calcium	Ca	2.20–2.65 mmol/l
Phosphate	Ph	0.78–1.53 mmol/l
Creatinine	Crea	57–113 μmol/l
Alkaline phosphatase	ALP	3–20.2 ng/ml
Parathyroid hormone	PTH	15–65 pg/ml

According to the available group size, only the serum vitamin D levels of the patients who did not receive supplementation were evaluated in quartiles. In addition, blood vitamin D levels were classified according to the recommendations of the German Society for Nutritional Medicine, as shown in Table 2.

**Table 2:** Definition of vitamin d status based on serum vitamin levels [13, 14]

Values in nmol/ml	Values in ng/ml	Category
< 12	< 4.80	Serious deficiency
12–30	4.80–12	Deficiency
30–50	12–20	Insufficient
50–75	20–30	Probable insufficient
75–100	30–40	Sufficient optimal
100–150	40–60	Probable too high
150–375	60–150	Hypervitaminosis
> 375	> 150	Toxic level

Patients were screened for osteoporosis and asked about previous screening tests. The osteoporosis assessment was performed according to the guideline of the DVO (Dachverband Osteologie, German Osteology Society) for the diagnosis of osteoporosis [7], which was valid at that time. This included a survey of existing risk factors and the baseline laboratory. Irrespective of vitamin D intake, the information from the questionnaire was used to determine whether there was an indication for a prior diagnosis of osteoporosis according to the DVO guideline. In addition, the patient was asked about laboratory tests that had been performed in the past and about a DXA measurement, as well as about the medications and dietary supplements that he or she was taking. It was also recorded whether, based on the risk factors assessed, the patient had an indication for a diagnosis of osteoporosis according to the DVO guideline prior to the current fracture. The assessment was completed by calculating the Fracture Risk Assessment Tool® (FRAX) [11] and classifying the patients according to the American Association of Clinical Endocrinologists (AACE) treatment recommendation [12]. In this way, patients with a fracture risk of three percent for hip fractures and 20 percent for major osteoporotic fractures were classified as having osteoporosis. To avoid bias from the current fracture, these values were determined without taking the current fracture into account.

The DVO guideline distinguishes between basic and specific therapies for osteoporosis with the substitution of vitamin D being part of the basic therapy. Specific therapy refers to either osteoanabolic or anti-resorptive therapy.

Descriptive data was analyzed using Microsoft Excel 2019. For further statistical analysis, SPSS version 28 was used. After testing for normal distribution using the Kolmogorov–Smirnov test, groups were examined using the Mann–Whitney *U* test for gender and the Kruskal–Wallis test for age, as the data was not normally distributed. *p* values less than 0.05 or greater than 0.95 were considered significant.

## Results

For this study, 102 patients who underwent surgery for a fracture of the distal radius at our hospital between April 2021 and April 2022 were prospectively enrolled. According to the inclusion criteria, the patients’ age ranged from 46 to 91 years. The sex ratio was approximately 1:5 with 17 males, only. Detailed demographic information is given in Table 3, as well as the blood values at the time of examination divided according to vitamin D intake. None of the patients in the study had a toxic vitamin D level.

**Table 3:** Characteristics of the included patients according to their vitamin d prescription/intake behavior (with self-prescribed (group 1), without (group 2) and with physician-prescribed (group 3) vitamin D supplementation)

	Group 1 (*n* = 12)	Group 2 (*n* = 83)	Group 3 (*n* = 7)
Age	66 (11)	70 (12)	73 (12)
Gender			
Female	12	66	7
Male	0	17	0
Serum level			
Vit. D in nmol/ml	99.9 (57.0)	49.6 (28.4)	72.1 (39.0)
Ca	1.9 (0.1)	1.9 (0.2)	1.9 (0.1)
Ph	1.3 (0.2)	1.1 (0.2)	1.1 (0.2)
Crea	79 (36)	71 (24)	81 (47)
ALP	12 (5)	14 (6)	17 (11)
PTH	43 (16)	63 (62)	75 (58)

Data are presented as mean values with standard deviation in brackets

**Fig. 1:**
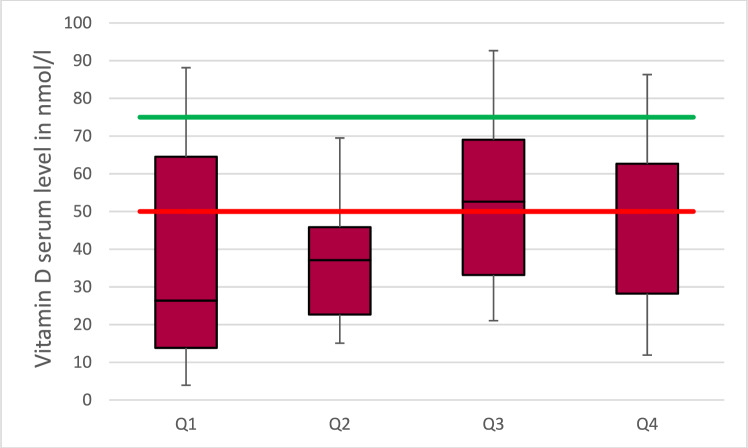
The box-plots show the average vitamin d level spread over the quarters of the year (Q1–Q4) in patients without vitamin d supplementation. The red line indicates the lower cut-off and the green line the value for the ideal level of vitamin D

In the group of patients not taking vitamin D supplements, there was no month in which the recommended serum level of 75 nmol/l was exceeded. A plot for the annual quarters is shown in Fig. 1. However, a seasonally significant difference (*p* = 0.022) was found when comparing the first and second quarter with the third quarter (*p* = 0.01 and *p* = 0.02, respectively).

In group 2, a distinction was now made according to sex. The mean vitamin D level for men (*n* = 17) was 42 nmol/l. In women this value was higher at 51 nmol/l without statistical significance (*p* = 0.194).

**Fig. 2:**
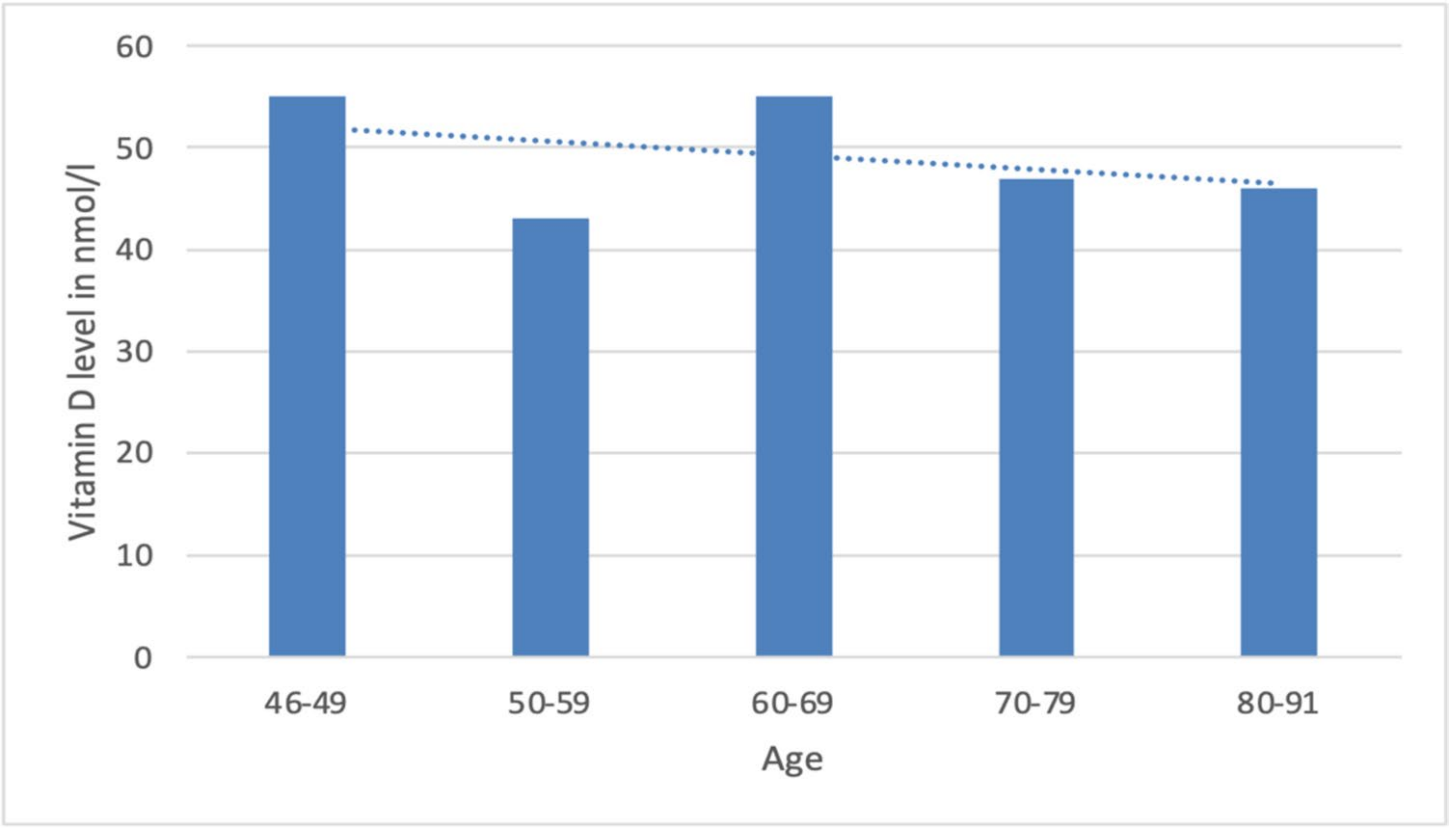
Plots of the mean serum vitamin D levels as a function of patient age. The dashed line is the linearly calculated trend line

According to age, patients in group 2 under 65 years of age (*n* = 33) had a mean vitamin D level of 54 nmol/l. Patients over 65 years of age had a mean value of 46 nmol/l. Figure 2 shows a more detailed breakdown of the values for each decade. The differences between the age groups were small (*p* = 0.690), but there was a trend of decreasing values with increasing age.

Of all patients, 75% (*n* = 76) met the DVO diagnostic criteria. This was the case for 82% (68 out of 76) of the female patients and for 47% (8 out of 17) of the male patients. Fifty-three percent of the patients in this category had never undergone a test for the diagnosis of osteoporosis.

Based on the 10-year fracture risk assessment, 57 patients had indications for treatment before experiencing the current fracture. Out of this group, 12 individuals had osteoporosis as a secondary diagnosis, and four of them, which constitute a third of the total, were receiving specific osteoporosis therapy. All these patients were female. Consequently, there is a treatment gap of 66% for patients diagnosed with osteoporosis as a secondary condition, and a treatment gap of no less than 84% for all patients.

Of the patients who showed signs of osteoporosis requiring treatment, four received specific therapy that included basic therapy. Nine received only basic therapy. None of the six male patients recommended to be treated with specific therapy received it. Considering the types of treatment administered, a shortfall of 84% was observed in basic therapy and 93% in specific therapy. For women, the percentages were 82% and 92%, respectively, while for men it was 100%.

## Discussion

Knowing the sentinel function of a distal radius fracture as an early manifest sign of osteoporosis, early diagnosis appears to be particularly important in this patient population. It is crucial for the patient's future quality of life and is the first step in preventing further osteoporosis-related fractures. Chen et al. [4] showed that the risk of hip fracture is five times higher after a radius fracture and Haentjens et al. [3] that the relative risk is increased by a factor of 1.5 in women and by a factor of 3.26 in men.

The aim of treatment of a distal radius fracture should be not only to treat the fracture, but also to identify and treat any associated bone metabolic disorders. Regardless of whether osteoporosis is present, vitamin D plays a key role in bone metabolism. This is also reflected in the fact that any deficiency should be corrected before starting specific osteoporosis therapy [7].

The results presented here demonstrate a diagnosis and treatment gap in this group of patients. This gap existed prior to the distal radius fracture. They also support the hypothesis that most elderly patients do not achieve adequate serum vitamin D levels. Although we were able to demonstrate a higher vitamin D level in the summer months, these levels still appeared to be too low for an adequate vitamin D supply. The patients were not able to build up a depot for the winter.

The diagnosis gap found in this study was 53% and the treatment gap was 84% for patients with distal radius fractures. These percentages are higher than those reported in the literature for the German population as a whole. In a European-wide evaluation, Kanis et al. [5] found a treatment gap of 76% for women in Germany. Even this was below the 82% treatment gap for women identified here. This highlights the vulnerability of fracture patients, in our opinion. The results for men were particularly alarming, showing once again that men are even less taken into account than women when it comes to the risk of osteoporosis [15, 16].

The vitamin D levels obtained were in line with those found in other studies for the total population, without focusing on patients with distal radius fractures [17]. Chinoy et al. [18] also looked at vitamin D levels in patients with distal radius fractures and reported a mean level of 37.5 nmol/ml, similar to that found in the Karachi patients. Similarly, in their study of Korean women, Jang et al. [19] found that patients with distal radius fractures had lower serum vitamin D level. Neither study examined the indication for osteoporosis diagnosis or treatment. To our knowledge, there are no trials that had calculated fracture risk and simultaneously analyzed the indication for diagnostic efforts of osteoporosis. Similarly, there are no analyses that evaluated vitamin D supplementation. Bias due to self-medication was significantly reduced here by classifying patients according to their vitamin D supplementation and taking into account the source of this supplementation.

Remarkably, all the men were in the group without vitamin D replacement. This suggests that this cohort may be underdiagnosed in terms of vitamin D status, and not just osteoporosis. According to the existing diagnostic criteria, almost half of the men from our study still had an indication for osteoporosis diagnostic. The lower proportion of men compared with women is probably due to the fact that, according to the guidelines, men enter the risk group for osteoporosis 10 years later than women [7]. Despite this, the 80% diagnosis gap observed for the total population was exceeded for male patients with distal radius fractures [5].

The seasonal course of vitamin D levels without substitution over the year can be understood very well. A seasonal difference in serum vitamin D levels has been reported differently in the literature. Stolarczyk et al. [20] and Jang et al. [19] found no seasonal difference in their patients. Bleizgys and Kurovski [17], Gannagé-Yard et al. [21] and Cabral et al. [22] were able to show a seasonal difference in their study with lower vitamin D levels in January and February and the highest levels in August. The latter was also reflected in our results, where vitamin D levels increased in the summer months, as expected. Despite the increase, sufficient levels could not be achieved without substitution. With a value of 50 nmol/l, the average value remains below the recommended value of at least 75 nmol/l. This should be of particular concern in view of the fact that vitamin D levels are still too low in the study population. This has to be seen in the context of the need to replenish vitamin D stores in the summer, as endogenous synthesis is insufficient in the winter [8]. By testing patients in the laboratory over a 12 month period, seasonal bias was avoided, and at the same time statements could be made about the development of vitamin D levels within a calendar year. This is another advantage of this study.

For the assessment according to gender, the results were described in different ways in the literature. Yan et al. [23] were able to show a significantly higher value for men compared to women in Chinese adults. The authors were also able to show that vitamin D levels were higher in women with increasing age. It should be noted that the average age of 49 years in the population studied was significantly younger than in this study. In contrast, Härdi et al. [24] were able to show higher vitamin D levels in female participants among the staff of a Swiss hospital. In a similar group of patients, Gennagé-Yard et al. [21] also showed a better value for women, but no correlation of vitamin D levels with age.

### Limitations

The study population is small, but corresponds to the number of patients in studies on the determination of vitamin D levels in patients with distal radius fractures [18–20, 25]. Another limitation is the documentation of the supplements, which was primarily derived from the patient survey and medication plan. Adequate information on vitamin D intake was not provided by all patients, and not all patients were taking all prescribed medications, which may have led to misclassification of vitamin D intake. Nevertheless, the authors are not aware of any previous attempts to take vitamin D intake into account, making this study the first of its kind. It is therefore certain to provide a more accurate picture than previous publications that did not take vitamin D intake into account. A potential bias may arise from the exclusion of conservatively treated fractures. The mean age was 70 years, consistent with the age range of 65 to 85 years with the highest incidence of distal radius fractures. The male to female ratio of 5:1 also matched the literature [2, 19]. Therefore, it can be inferred that the examined sample was representative for the entire patient population with distal radial fractures.

The fact that it was not possible to determine how much time had elapsed since the previous diagnosis may explain the comparatively lower percentage of the diagnosis gap compared to the treatment gap. This includes an inaccuracy for patients who had a negative diagnosis of osteoporosis in the past and who have since developed an additional risk factor that would have required a new diagnosis. In addition, the diagnostic gap may be smaller because this study also considered a laboratory test or DXA scan alone as a diagnosis. This was done to account for the fact that the DVO guidelines do not require both to be done in all cases.

## Conclusions

The summary of this analysis showed that the majority of our patients with osteoporosis-associated distal radius fractures had an indication for further diagnosis of osteoporosis prior to the fracture, and half of them had an indication for specific therapy especially for men. The study also showed that almost all patients were in the range of vitamin D deficiency or insufficiency and that serum vitamin D levels should preferable be measured in all fracture patients. It was also striking that patients with self-prescribed vitamin D supplementation had the best serum levels. This suggests a widespread chronic undersupply of vitamin D, which also extends to the group of patients on substitution. Therefore, the serum levels of these patients should also be checked regularly. The data also suggests that vitamin substitution should be considered throughout the year in all our fracture patients. This recommendation is not only in the sense of basic osteoporosis therapy, but also in the sense of prevention of osteomalacia, which also benefits patients who do not actually have manifest osteoporosis.

## Data Availability

All data on which the conclusions of the paper rely are included in this published article.
